# Nevus lipomatosus cutaneous superficialis*

**DOI:** 10.1590/abd1806-4841.20164570

**Published:** 2016

**Authors:** Gustavo de Sá Menezes Carvalho, Silvana Maria de Morais Cavalcanti, Alzinira Souza Herênio, Márcia Almeida Galvão Teixeira, Eliane Ruth Barbosa de Alencar, Sergio Paulo Mendes Gonçalves

**Affiliations:** 1 Universidade de Pernambuco (UPE) – Recife (PE), Brazil

**Keywords:** Adipocytes, Skin abnormalities, Nevus, Skin

## Abstract

We report a case of nevus lipomatosus cutaneous superficialis of Hoffman-Zurhelle (NCLS), with multiple lesions, in a ten-year-old child. The NLCS is considered rare. The classical clinical presentation is characterized by multiple skin-colored or yellowish papules and nodules, which can have a linear distribution. Histologically, it is characterized by the presence of mature ectopic adipocytes in the dermis. The main therapeutic option is surgical excision. The classical Nevus lipomatosus cutaneous superficialis is reported in this case.

## INTRODUCTION

Nevus lipomatosus cutaneous superficialis (NLCS) is a rare entity. It was first described by Hoffman and Zurhelle in 1921.^1^ According to the literature (LILACS, IBECS, MEDLINE, Biblioteca Cochrane, SciELO), the the latest case in Brazil was published in 1999 by Almeida et al. Clinically, the disease is characterized by multiple non-painful pedunculated, cerebriform, yellowish or skin-colored papules or nodules, which can have a linear distribution.^1,2^ Histologically, it is characterized by the presence of mature ectopic adipocytes in the dermis. We report a linear variant of NLCS in a child.

## CASE REPORT

**Figure 1:**
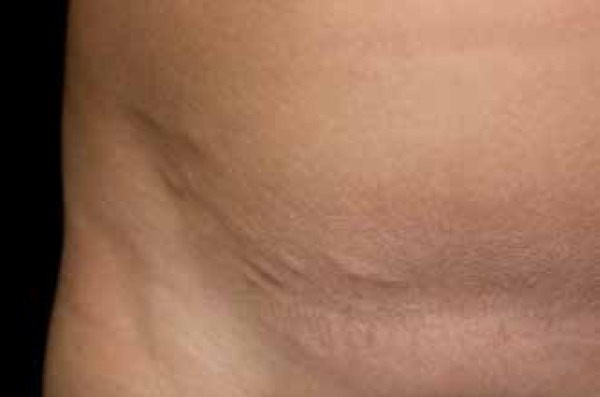
Clinical appearance of the NLCS: papules in linear distribution located in the right inguinal region

**Figure 2:**
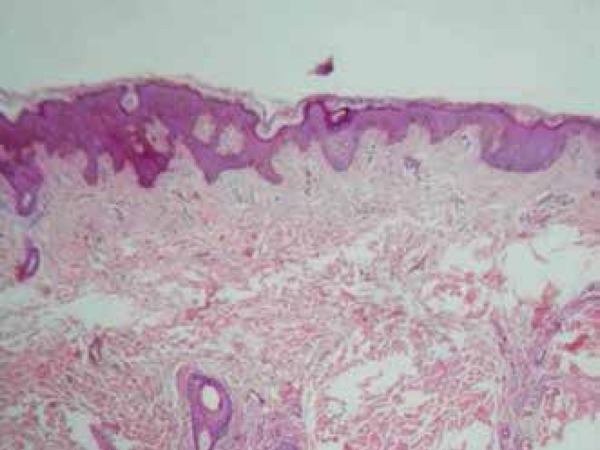
Deposition of adipose tissue in the dermis (HE x10)

**Figure 3:**
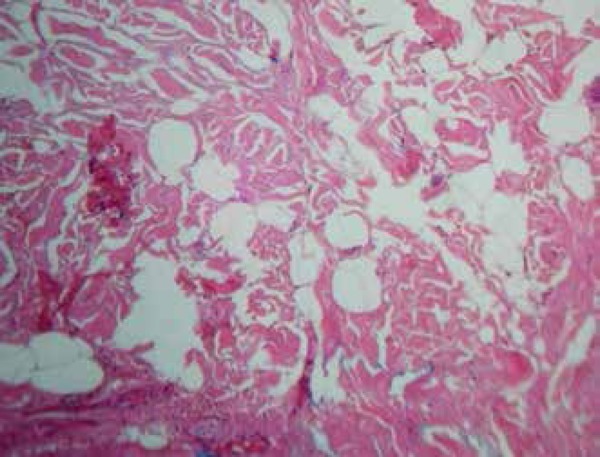
Mature ectopic adipocytes grouped in irregular strips between the collagen bundles of the reticular dermis (HE x40)

We report a ten-year-old mulatto patient with a history of asymptomatic lesions, which had been progressing since the age of two. Dermatological examination revealed skin-colored plaque lesions, irregular surface, on the left arm, in the right inguinal region, mesogastric region, and on left flank in a linear distribution (Figure 1). Histopathological examination showed mature ectopic adipocytes grouped into irregular strips between the collagen bundles of the reticular dermis, consistent with the diagnosis of NLCS (Figures 2 and 3, slide).

## DISCUSSION

NLCS is characterized by multiple yellowish or skin-colored papules or nodules. They can affect the pelvis, lumbosacral region, and butocks.^3^ They are rarely located in other areas. Festa Neto et al. (1984) reported the first case of NLCS in Brazil in a 4-year-old patient with multiple hairy lesions on the wrist.^4^

NLCS exists in two clinical varieties: 1) the solitary form, with a single lesion and variable location; 2) the linear classic form, with multiple lesions in the pelvic and gluteal regions, as described by Hoffman and Zurhelle.^1^ Its clinical aspect may vary from lobulated tumor lesions – with a pleated cerebriform appearance – to small neoformations covered by normal skin – producing just a slight elevation that may go unnoticed. Occasionally, these lesions can present with hair growth.^5,6^ The present case was characterized by multiple lesions with a linear distribution.

The pathogenesis of NLCS is still not well defined. Jones (1975) suggested that ectopic adipocyte would originate from precursor cells from the dermal vessels.^6^ Other authors consider the hypothesis that the deposition of adipocytes is caused by degenerative alterations of dermal collagen bundles and elastic tissue.^6,7^

Histopathologically, there is, in the superficial and middle layers of the dermis, the presence of dispersed or grouped lobules of adipose tissue, composed of well-differentiated adipocytes – preferably in a perivascular distribution – and surrounded by collagen fibers with normal appearance.^7,8^

Clinical differential diagnosis of the NLCS includes other tumors or nevi that may be linear or multiple and zosteriform in distribution, such as: connective nevus, sebaceous nevus and verrucous nevus.^9^ Our patient had lesions that clinically resembled connective nevus. However, histopathology demonstrated the presence of adipocytes in the dermis, confirming the diagnosis of NLCS. The therapeutic option for this nevus is surgical excision, with no reports of other forms of treatment.^2,10^
